# Exploring the photoleakage current and photoinduced negative bias instability in amorphous InGaZnO thin-film transistors with various active layer thicknesses

**DOI:** 10.3762/bjnano.9.239

**Published:** 2018-09-26

**Authors:** Dapeng Wang, Mamoru Furuta

**Affiliations:** 1 Key Laboratory of Applied Surface and Colloid Chemistry, Ministry of Education; Shaanxi Key Laboratory for Advanced Energy Devices; Shaanxi Engineering Lab for Advanced Energy Technology, School of Materials Science and Engineering, Shaanxi Normal University, Xi’an 710119, China; 2 School of Environmental Science and Engineering, Kochi University of Technology, Kami, Kochi 782-8502, Japan; 3 Center for Nanotechnology in Research Institute, Kochi University of Technology, Kami, Kochi 782-8502, Japan

**Keywords:** active layer thickness, gate bias, illumination stress, InGaZnO, photoleakage current, thin-film transistors

## Abstract

The photoleakage current and the negative bias and illumination stress (NBIS)-induced instability in amorphous InGaZnO thin-film transistors (a-IGZO TFTs) with various active layer thicknesses (*T*_IGZO_) were investigated. The photoleakage current was found to gradually increase in a-IGZO TFTs irrespective of the *T*_IGZO_ when the photon energy of visible light irradiation exceeded ≈2.7 eV. Furthermore, the influence of the *T*_IGZO_ on NBIS-induced instability in a-IGZO TFTs was explored by the combination of current–voltage measurements in double-sweeping *V*_GS_ mode and capacitance–voltage measurements. The NBIS-induced hysteresis was quantitatively analyzed using a positive gate pulse mode. When the *T*_IGZO_ was close to the Debye length, the trapped electrons at the etch-stopper/IGZO interface, the trapped holes at the IGZO/gate insulator interface, and the generation of donor-like states in an a-IGZO layer were especially prominent during NBIS.

## Introduction

Over the last decade, the amorphous oxide-based semiconductor thin-film transistors (AOS TFTs) have attracted global attention for use in advanced display technologies due to their outstanding properties such as high electron mobility, good transparency to visible light, and low process temperature with good uniformity [1–4]. Among the numerous AOS materials, indium gallium zinc oxide (IGZO) is one of the most promising candidates used as the active layer because of its excellent electrical and optical properties [5–8]. Although the band gap of IGZO (≈3.1 eV) is higher than the photon energy of visible light, photoinduced leakage current under visible-light irradiation can be detected in the oxide-based TFTs [9–10]. This is due to the fact that the electrons are excited from the trapped states existing near the valence band (*E*_V_). In addition, the a-IGZO TFTs inevitably suffer electrical and optical stresses during practical operation conditions, especially for the negative bias and illumination stress (NBIS) tests [11–16], which leads to device instability and restricts the development of oxide TFTs for commercial products.

In our previous study, a double-sweeping *V*_GS_ mode was proposed to investigate the origin of NBIS-induced hysteresis of a-IGZO TFTs [17]. A promising method to suppress NBIS degradation was also considered by applying a large negative *V*_DS_ bias of *V*_DS_ < *V*_GS_ during NBIS [18]. These studies imply that the fabrication parameters for the active layer should be well taken into account to improve the reliability of oxide TFTs. The active layer thickness is a key parameter to modify the performance of a-IGZO TFTs. Some works have highlighted that the electrical properties of the device (for both the initial and after stress conditions) such as threshold voltage, on/off ratio, and field effect mobility, can be effectively adjusted by controlling the active layer thickness [19–23]. Up to now, the impact of the active layer thickness (*T*_IGZO_) on the photoleakage current and NBIS-induced instability in a-IGZO TFTs has been rarely reported. The NBIS-induced degradation of a-IGZO TFTs with various active layer thicknesses has also rarely been discussed.

In this study, a-IGZO films with various active layer thicknesses were prepared by magnetron sputtering. The initial electrical properties and the photoleakage current of a-IGZO TFTs with various active layer thicknesses were investigated. The subthreshold value slightly increased while the threshold voltage (*V*_th_) and mobility (μ) decreased with increasing *T*_IGZO_. The photoleakage current increased in all TFTs when the wavelength of visible-light irradiation was shorter than 460 nm. Moreover, the photoleakage current increased with an increase in the *T*_IGZO_. Furthermore, the impact of the active layer thickness on the NBIS-induced instability in a-IGZO TFTs was explored by combining the current–voltage (*I*–*V*) measurements in double-sweeping *V*_GS_ mode and capacitance–voltage (*C*–*V*) measurements. The NBIS-induced hysteresis was quantitatively analyzed using a positive gate pulse mode. The *I*–*V* and *C*–*V* results revealed that the trapped holes at the etch-stopper/IGZO interface, the trapped holes at the IGZO/gate insulator interface, and the generation of donor-like states in a-IGZO layer were particularly prominent after NBIS tests when the active layer thickness was close to the Debye length.

## Experimental

**Figure 1:**
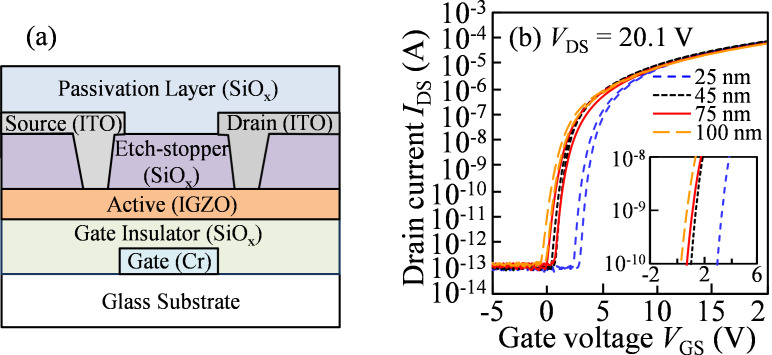
(a) Schematic cross-sectional view and (b) the initial transfer characteristics of a-IGZO TFTs with various active layer thicknesses (*T*_IGZO_) measured at *V*_DS_ = 20.1 V.

A schematic cross-sectional view of a bottom-gate a-IGZO TFT is shown in Figure 1a. The detailed fabrication procedure for the a-IGZO TFT was described in our previous publication [24]. After the fabrications of a chromium (Cr) gate electrode and a SiO*_x_* gate insulator (150 nm), the a-IGZO layer with thicknesses of 25, 45, 75, and 100 nm respectively were deposited at 160 °C from a sintered IGZO ceramic target by DC magnetron sputtering with a mixed gas of Ar/O_2_ = 29.4/0.6 sccm at a deposition pressure of 1 Pa. After patterning of the IGZO films as the active channel, a SiO*_x_* etch-stopper (200 nm), source and drain electrodes, and a 200 nm-thick SiO*_x_* passivation layer were sequentially formed. Following the preparation of a-IGZO TFTs, all devices were annealed in N_2_ environment at 350 °C for 1 h before the electrical measurements. The channel width (*W*) and length (*L*) of the IGZO TFTs were 50 and 20 μm, respectively. All of the *I*–*V* characteristics were measured using an Agilent 4156C precision semiconductor parameter analyzer.

For the photoleakage current test, monochromatic light irradiation was supplied by a Xe lamp with a band pass filter (FWHM of 10 nm) at an intensity of 0.2 mW/cm^2^. The wavelength of the light was in the range of 400–530 nm and was introduced to the back-channel side of a-IGZO TFTs. For the NBIS test, blue light with a wavelength of 460 nm and a gate voltage (*V*_GS_) of −30 V was simultaneously applied to all devices during the stress test. The NBIS was interrupted briefly when the transfer characteristics were measured in double-sweeping *V*_GS_ mode in darkness at *V*_DS_ = 0.1 V, and then NBIS was reapplied up to a stress time of 10^4^ s. In terms of the double-sweeping *V*_GS_ mode, the transfer characteristics were measured with *V*_GS_ = −10–20 V (denoted hereafter as forward measurement), and then scanned instantly back to −10 V (denoted hereafter as reverse measurement). The *C*–*V* measurements for the channel capacitance were measured at 1 kHz and an AC level of 100 mV. All of the measurements were carried out at room temperature in ambient air.

## Results and Discussion

The initial transfer characteristics (*I*_DS_–*V*_GS_) of a-IGZO TFTs with various active layer thicknesses (*T*_IGZO_) measured at *V*_DS_ = 20.1 V are shown in Figure 1b. Table 1 summarizes the electrical properties, such as field effect mobility in the saturation region (μ_sat_), threshold voltage *V*_th_ (*V*_GS_ at *I*_DS_ of 1 nA), hysteresis of the transfer curves (the difference of *V*_GS_ at *I*_DS_ of 1 pA between the forward and reverse sweeps), subthreshold swing (SS = d*V*_GS_/dlog_10_(*I*_DS_)), and the maximum area density of state (*N*_t_).

**Table 1:** The initial electrical properties of a-IGZO TFTs with various active layer thicknesses (*T*_IGZO_).

Thickness, *T*_IGZO_ (nm)	25	45	75	100

μ_sat_ (cm^2^∙V^−1^∙s^−1^)	13.61	13.47	12.43	12.20
*V*_GS_ at *I*_DS_ = 1 nA (V)	3.35	1.24	1.16	0.74
hysteresis Δ*V*_H_ (V)	0.47	0.42	0.35	0.30
subthreshold swing (mV/dec.)	264	273	316	352
trap density (10^11^ cm^−2^)	5.06	5.29	6.35	7.24

Compared to a previous publication [25], the electrical properties of a-IGZO TFTs with various *T*_IGZO_ exhibit the identical tendency. It is suggested that the devices exhibit great repeatability for the same kind of material under the same fabrication process. In addition, the *V*_th_ results demonstrate that the free carrier numbers in the bulk of the active layer are gradually increased with increasing *T*_IGZO_. Moreover, since the fabrication condition for the IGZO films are exactly identical, except the deposition duration, the results suggest that the variation in the SS value mainly originates from the density of defect states in the active layer. Correspondingly, the obtained results indicate that the increase in the *N*_t_ majorly stems from the increase of the IGZO bulk traps because of the identical a-IGZO/GI interfaces.

**Figure 2:**
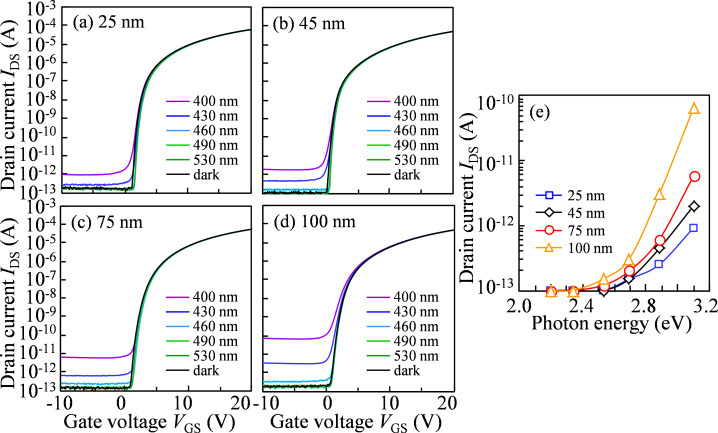
Variation in the transfer characteristics of a-IGZO TFTs with (a) *T*_IGZO_ = 25, (b) 45, (c) 75, and (d) 100 nm under monochromatic light irradiation, and (e) photoleakage current (*V*_GS_ = −10 V and *V*_DS_ = 10 V) of a-IGZO TFTs with various thicknesses as a function of photon energy of the incident light.

Figure 2a–d shows the variation in the transfer characteristics of a-IGZO TFTs with various *T*_IGZO_ under monochromatic light irradiation at the excitation wavelengths of 400, 430, 460, 490, and 530 nm. The transfer characteristics measured in the dark are also shown as a reference. The transfer characteristics of all devices are measured with a *V*_DS_ of 10.1 V and a *V*_GS_ scanned from the on-to-off direction. It is found that the photoleakage current increases in all TFTs when the irradiation wavelength is shorter than 460 nm. In addition, the photoleakage current increases with increasing *T*_IGZO_. Figure 2e exhibits the photoleakage current of a-IGZO TFTs with various *T*_IGZO_ as a function of the photon energy of incident light. When the photon energy exceeds ≈2.7 eV (460 nm), the photoleakage current starts to increase and increases gradually with increasing photon energy. Note that the photoleakage current increases dramatically in the photon energy range of >2.7 eV for the TFT with the thicker *T*_IGZO_. These results indicate that the electrons are excited from the trapped states existing near the valence band (*E*_V_) to the conduction band (*E*_C_) even though the photon energy is smaller than the band gap of IGZO. In terms of the a-IGZO material, the high-density electron traps exist at (*E*_C_ − *E*) of over 2.7 eV [9], which affect the photoleakage current of a-IGZO TFTs. The total amount of trapped electrons increase with an increase in the *T*_IGZO_. The oxygen-related defects, such as oxygen vacancies (V_O_), may be the origin of high-density electron traps near the *E*_V_ in a-IGZO TFTs, which occupy the region near the valence band maximum with an energy width of ≈1.5 eV [26–27].

**Figure 3:**
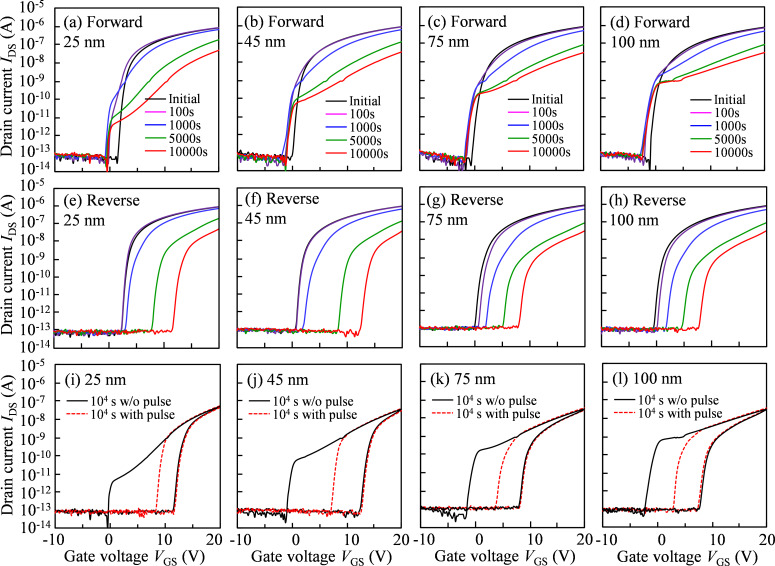
Variation in the transfer characteristics in the forward measurement for a-IGZO TFTs with (a) *T*_IGZO_ = 25, (b) 45, (c) 75, and (d) 100 nm, and in the reverse measurement with (e) *T*_IGZO_ = 25, (f) 45, (g) 75, and (h) 100 nm as a function of the stress duration for 10^4^ s under −30 V *V*_GS_ NBIS. Transfer characteristics of a-IGZO TFTs with (i) *T*_IGZO_ = 25, (j) 45, (k) 75, and (l) 100 nm after 10^4^ s NBIS without and with a gate pulse of 1 ms width and 10 V high.

Figure 3a–d shows the variation in the transfer characteristics of a-IGZO TFTs with various *T*_IGZO_ under NBIS for the forward measurements. It is found that for the NBIS duration of 1000 s, the transfer curves of all TFTs shift in the negative *V*_GS_ direction without SS degradation. When the NBIS duration exceeds 1000 s, a shift in the positive *V*_GS_ direction as well as the appearance of a hump with SS degradation in the transfer curves is observed, and this phenomenon gradually increases with increasing NBIS duration. It is noted that the phenomenon, combined the positive shift and the hump effect, is weakened as the *T*_IGZO_ increases. For the reverse measurements, as shown in Figure 3e–h, the transfer curves of all TFTs shift parallel in the positive *V*_GS_ direction without SS degradation during the NBIS duration of >1000 s. Noticeably, the abnormal phenomenon of hump appearance observed in the forward measurements is hardly observed in the reverse measurements. The positive shift of *V*_th_ without SS degradation is well fitted to the commonly used stretched-exponential equation [28]. The obtained results suggest that electron trapping at the back-channel interface between a-IGZO and etch-stopper layers occurs because a negative gate bias is performed during NBIS.

On the basis of the photoleakage current results, when the photon energy of the light irradiation exceeds ≈2.7 eV, the photoleakage current of TFTs increases. In this study, a photon energy of ≈2.7 eV is set for the incident light. The previous publication indicates that a photon energy of ≈2.0 and ≈2.3 eV is required for the transition from V_O_ to V_O_^+^ and V_O_^2+^, respectively. Moreover, the ionized oxygen vacancies of V_O_^+^ and V_O_^2+^ are located near the mid-gap and the bottom of the *E*_C_ [15,29], respectively. It is sufficient to excite high-density V_O_ defects to V_O_^+^/V_O_^2+^ and then to generate free electrons to *E*_C_. Simultaneously, the electron–hole pairs are photoexcited from *E*_V_, which leads to the neutralization between the ionized V_O_^+^/V_O_^2+^ and the generated electrons, contributing to free holes in *E*_V_ [18]. During the NBIS duration with *V*_GS_ = −30 V, a vertical electric field is exerted along the growth direction of the active layer. In general, the electric potential exponentially declines inside the active layer and has a maximum transfer length called the Debye length. In terms of a-IGZO TFT, a Debye length of ≈40 nm is calculated based on a previous publication [30]. In case of a-IGZO TFT with the *T*_IGZO_ = 25 nm, the channel layer is totally depleted under the negative *V*_GS_ bias since the *T*_IGZO_ is less than the Debye length. Therefore, the photoexcited electrons and holes will be respectively accumulated and trapped at the IGZO/etch-stopper and the GI/IGZO interfaces. Meanwhile, the defect states are generated, which originate from the photoexcited V_O_^+^/V_O_^2+^. In the forward measurement, the transfer curves exhibit a positive shift in the *V*_GS_ direction with a hump at the turn-on voltage region when the NBIS duration exceeds 1000 s, which is attributed to the synergistic effects of the generated defect states and the trapped holes at the front-channel interface. After the forward measurement with *V*_GS_ = −10–20 V, the ionized V_O_^+^/V_O_^2+^ would be gradually neutralized by capturing electrons, and the trapped holes at the front-channel interface are completely de-trapped due to the vertical electric field induced by the positive *V*_GS_. Consequently, the abnormal hump observed in the forward measurement disappears in the reverse measurement, suggesting that the donor-like defect states, located near the Fermi level (*E*_F_) at *V*_GS_ of the turn-on voltage, are generated and stabilized in the IGZO layer. It is noted that the trapped electrons at the back-channel interface are hardly de-trapped even when the positive *V*_GS_ is applied [17]. As a result, the transfer curves in the reverse measurement exhibit a parallel shift of 10.03 V without SS degradation in the positive *V*_GS_ direction after the NBIS duration of 10^4^ s.

**Figure 4:**
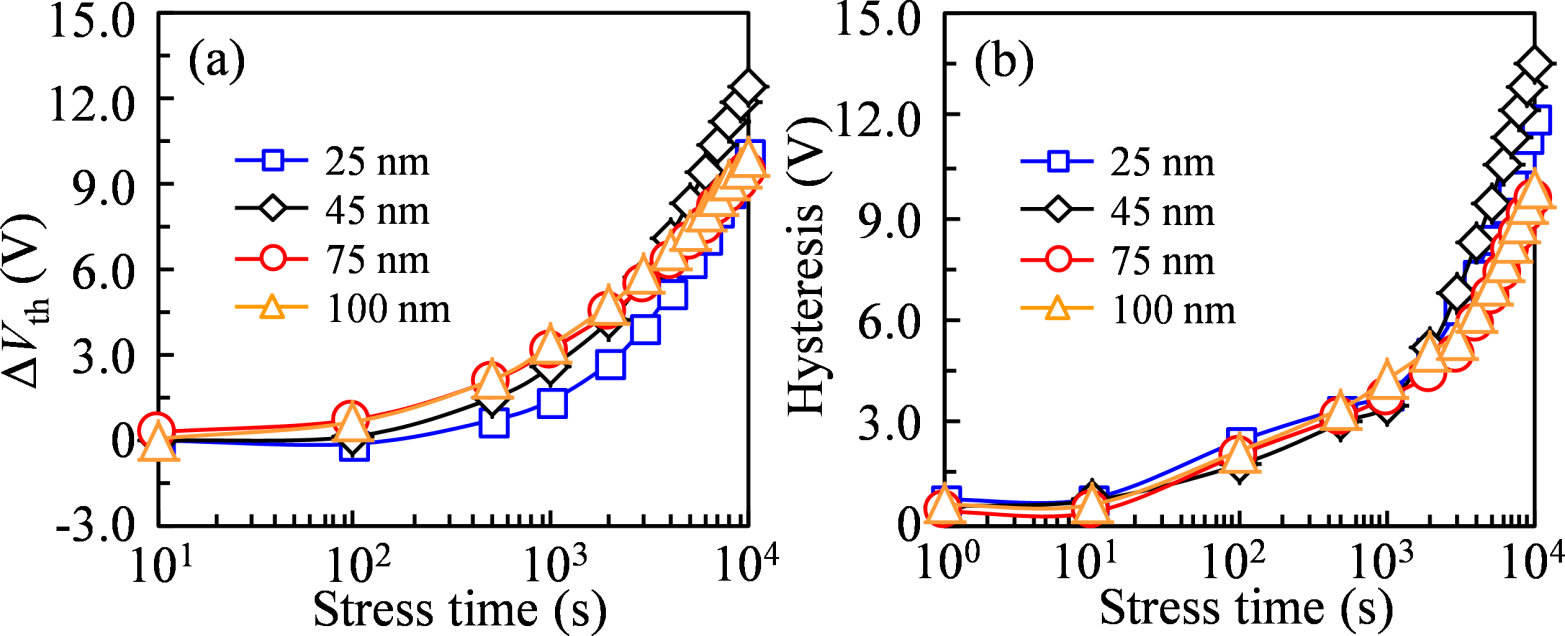
(a) Variation in *V*_th_ of the transfer curves of a-IGZO TFTs with various *T*_IGZO_ in the reverse measurement as a function of NBIS duration and (b) hysteresis of a-IGZO TFTs with various *T*_IGZO_ as a function of NBIS duration.

When the *T*_IGZO_ is increased to 45 nm, which is close to the Debye length, the whole channel layer is almost depleted under the negative *V*_GS_ bias. During the NBIS duration, more electrons and holes are excited and trapped at the back-channel and the front-channel interfaces. Simultaneously, the high-density defect states are generated due to the increase in the photoexcited V_O_^+^/V_O_^2+^. As a consequence, after the 10^4^ s NBIS duration, the transfer curves show a significant shift in the positive *V*_GS_ direction with a prominent hump for the forward measurement and display a distinct change of 12.46 V in the positive *V*_GS_ direction without SS degradation for the reverse measurement, as shown in Figure 4a. When the *T*_IGZO_ is further increased to 75 and 100 nm, which is larger than the Debye length, the electric potential exponentially decreases inside the active layer under −30 V *V*_GS_ bias. Although some amount of electrons are photoexcited to *E*_C_, they are partly accumulated and trapped at the back-channel interface due to the weaker vertical electric field. As a result, the excited hole in *E*_V_ and the ionized V_O_^+^/V_O_^2+^ near *E*_F_ at *V*_GS_ of the turn-on voltage would be neutralized by the free electrons. Therefore, after the NBIS duration of 10^4^ s, the transfer curves exhibit a small shift in the positive *V*_GS_ direction with a weak hump for the forward measurement, and show the small shift of 9.45 and 9.96 V in the positive *V*_GS_ direction for the reverse measurement corresponding to *T*_IGZO_ = 75 and 100 nm, respectively.

The combination of the transfer curves in the forward and reverse measurements after the NBIS duration of 10^4^ s is shown in Figure 4b. The NBIS-induced hysteresis increases remarkably from 11.94 V for the TFT with *T*_IGZO_ = 25 nm to 13.47 V for the TFT with *T*_IGZO_ = 45 nm, and decreases drastically to 9.54 and 9.93 V when the *T*_IGZO_ further increases to 75 and 100 nm. For further quantitative analysis of the origin of the NBIS-induced hysteresis, a positive gate pulse mode is carried out just after the NBIS duration of 10^4^ s. Based on our previous publication [17], the optimized condition of a positive gate pulse with pulse width of 1 ms and pulse height of 10 V is enough to neutralize the ionized V_O_^+^/V_O_^2+^-induced donor-like defect states while it has no influence on the trapped holes at the front-channel interface, as shown in Figure 3i–l. It is found that the trapped hole-induced hysteresis are 3.41, 5.58, 4.39, and 4.62 V corresponding to the IGZO TFTs with *T*_IGZO_ = 25, 45, 75, and 100 nm, respectively.

**Figure 5:**
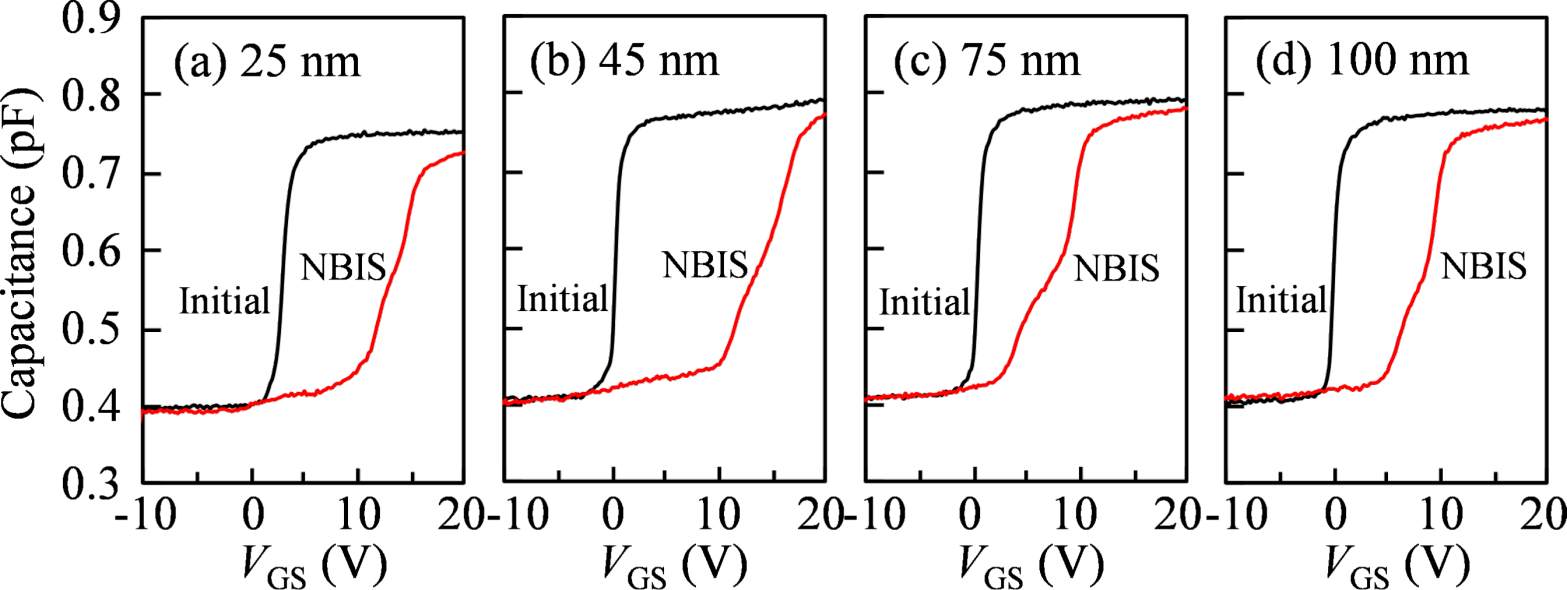
*C*−*V* curves before and after the NBIS duration of 10^4^ s with (a) *T*_IGZO_ = 25, (b) 45, (c) 75, and (d) 100 nm, respectively.

**Figure 6:**
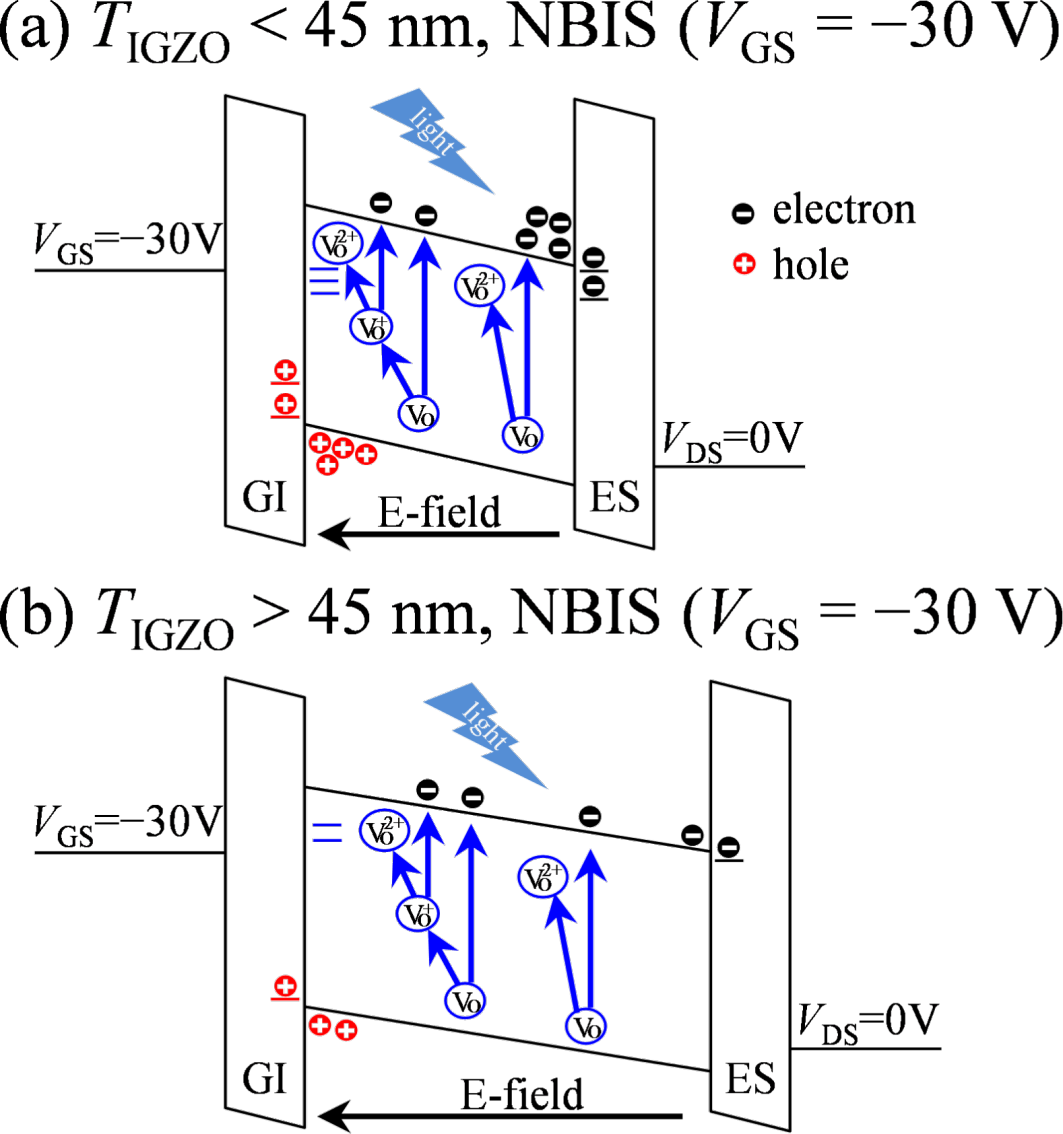
Schematic diagram of NBIS-induced degradation mechanism in a-IGZO TFTs with (a) *T*_IGZO_ < 45 and (b) over the Debye length (75 and 100 nm).

To further reveal the mechanism of the NBIS-induced hump and transfer curve shift in a-IGZO TFTs with various *T*_IGZO_, *C*–*V* analyses before and after the NBIS duration of 10^4^ s are measured, as shown in Figure 5. In the initial stage, all *C*–*V* curves without distortion are observed, which are in agreement with the initial *I*–*V* curves. After the 10^4^ s NBIS duration, all *C*–*V* curves shift in a positive *V*_GS_ direction with distortion near the turn-on region. The *C*–*V* results suggest that NBIS-induced defect states are uniform in the whole channel layer near *E*_F_ at *V*_GS_ of the turn-on voltage. In case of a-IGZO TFT with *T*_IGZO_ = 25 nm, the *C*–*V* curve shifts 11.4 V in the positive *V*_GS_ direction with a hump in the off-state. On the basis of the *C*–*V* results, the energy-band diagrams for the IGZO TFTs with various *T*_IGZO_ under NBIS are illustrated in Figure 6. The energy band at the front-channel is remarkably bent upward under the negative *V*_GS_ bias when the *T*_IGZO_ is less than the Debye length, as shown in Figure 6a. A hump observed at the turn-on region of the *C*–*V* curve indicates that the energy level of the generated defect states is located near *E*_F_ at *V*_GS_ of the turn-on voltage. The positive shift of the *C*–*V* curve demonstrates that electrons are trapped at the back-channel interface due to the vertical electric fields in the channel. When the *T*_IGZO_ is increased to 45 nm, the *C*–*V* curve exhibits a large shift of 15.1 V in the positive *V*_GS_ direction with a distinct hump at the turn-on region. The obtained results suggest that because the *T*_IGZO_ is close to the Debye length, the high-density defect states are generated in the whole channel layer and more electrons are photoexcited and trapped at the back-channel interface. When the *T*_IGZO_ is further increased to 75 and 100 nm, the *C*–*V* curves exhibit smaller shifts of 8.7 and 9.3 V in the positive *V*_GS_ direction with a weaker hump in the off-state compared to the 45 nm-thick channel layer case. The energy band at the front-channel is slightly bent upward as the *T*_IGZO_ is much larger than the Debye length, as shown in Figure 6b. The weakened hump near the turn-on region illustrates that the photoexcited V_O_^+^/V_O_^2+^ would be neutralized by the free electrons in *E*_C_, contributing to the low-density defect states near *E*_F_ at *V*_GS_ of turn-on voltage. The small shift of the *C*–*V* curves demonstrates that the fewer electrons are accumulated and trapped at the back-channel interface. The obtained *C*–*V* results are correlated with the results of the *I*–*V* measurements.

On the basis of the above discussion, it is demonstrated that the *T*_IGZO_ is one of the critical parameters to modify the electrical properties of the device. Besides the active layer thickness, the intrinsic characteristics of a-IGZO and the front- and back-channel interfaces of the TFT also play a vital role for the high-performance devices. Moreover, to reduce the density of oxygen vacancies in the bulk of the IGZO for the enhancement of electrical properties and stress stability of the TFTs, the following two aspects should be mainly considered: (i) oxidizing the densities of the defect state of oxide semiconductors to suppress charge trapping, for example by oxygen annealing and N_2_O plasma treatment [31]; and (ii) inactivating the defects in the semiconductor by means of introducing new elements to form stable chemical bonds with the defects, for example by fluoride ion implantation and nitrogen annealing [32–33].

## Conclusion

The impact of the *T*_IGZO_ on the photoleakage current and the NBIS-induced instability in a-IGZO TFTs were systematically investigated. It was found that when the photon energy of the light irradiation exceeds ≈2.7 eV, the photoleakage current increases in all TFTs irrespective of the *T*_IGZO_ due to the high-density electron traps existing at an (*E*_C_ − *E*) of ≈2.7 eV. Because the total amount of trapped electrons increases with increasing *T*_IGZO_, the photoleakage current gradually increases with increasing *T*_IGZO_. On the basis of the photoleakage current results, the influence of the *T*_IGZO_ on NBIS with a photon energy of ≈2.7 eV in a-IGZO TFTs is clarified by the *I*–*V* and *C*–*V* measurements. In addition, the NBIS-induced hysteresis is quantitatively evaluated through a positive gate pulse mode, contributing to the separation of the trapped holes at the front-channel interface and the generation of donor-like defect states in a-IGZO layer. The obtained *I*–*V* and *C*–*V* results indicate that when the *T*_IGZO_ is close to the Debye length, the trapped holes at the front-channel interface, the trapped electrons at the back-channel interface, and the generated donor-like defect states in a-IGZO are distinctly prominent during NBIS. This study suggests that to improve the reliability of oxide TFTs under light irradiation and gate bias stresses, the quality of the active layer and interface engineering should be taken into account.
